# The Concept, Development, and Application of a Home-Based High-Definition tDCS for Bilateral Motor Cortex Modulation in Migraine and Pain

**DOI:** 10.3389/fpain.2022.798056

**Published:** 2022-02-07

**Authors:** Alexandre F. DaSilva, Abhishek Datta, Jaiti Swami, Dajung J. Kim, Parag G. Patil, Marom Bikson

**Affiliations:** ^1^Headache and Orofacial Pain Effort Lab, Department of Biologic and Materials Sciences and Prosthodontics, University of Michigan School of Dentistry, Ann Arbor, MI, United States; ^2^Soterix Medical Inc., New York, NY, United States; ^3^Neural Engineering Laboratory, Department of Biomedical Engineering, The City College of New York, New York, NY, United States; ^4^Department of Neurosurgery, University of Michigan, Ann Arbor, MI, United States; ^5^Department of Neurology, University of Michigan, Ann Arbor, MI, United States; ^6^Department of Biomedical Engineering, University of Michigan, Ann Arbor, MI, United States; ^7^Department of Anesthesiology, University of Michigan, Ann Arbor, MI, United States

**Keywords:** tDCS, high-definition, chronic pain, home-based, bilateral stimulation, M1 stimulation, migraine

## Abstract

Whereas, many debilitating chronic pain disorders are dominantly bilateral (e.g., fibromyalgia, chronic migraine), non-invasive and invasive cortical neuromodulation therapies predominantly apply unilateral stimulation. The development of excitatory stimulation targeting bilateral primary motor (M1) cortices could potentially expand its therapeutic effect to more global pain relief. However, this is hampered by increased procedural and technical complexity. For example, repetitive transcranial magnetic stimulation (rTMS) and 4 × 1/2 × 2 high-definition transcranial direct current stimulation (4 × 1/2 × 2 HD-tDCS) are largely center-based, with unilateral-target focus—bilateral excitation would require two rTMS/4 × 1 HD-tDCS systems. We developed a system that allows for focal, non-invasive, self-applied, and simultaneous bilateral excitatory M1 stimulation, supporting long-term home-based treatment with a well-tolerated wearable battery-powered device. Here, we overviewed the most employed M1 neuromodulation methods, from invasive techniques to non-invasive TMS and tDCS. The evaluation extended from non-invasive diffuse asymmetric bilateral (M1-supraorbital [SO] tDCS), non-invasive and invasive unilateral focal (4 × 1/2 × 2 HD-tDCS, rTMS, MCS), to non-invasive and invasive bilateral bipolar (M1-M1 tDCS, MCS), before outlining our proposal for a neuromodulatory system with unique features. Computational models were applied to compare brain current flow for current laboratory-based unilateral M1^1^ and bilateral M1^2^ HD-tDCS models with a functional home-based M1^1−2^ HD-tDCS prototype. We concluded the study by discussing the promising concept of bilateral excitatory M1 stimulation for more global pain relief, which is also non-invasive, focal, and home-based.

## Introduction

Pain perception and sensitivity are generally considered adaptive for our survival. However, whatever the sources, some pain conditions become chronic, even with no clear cause (e.g., tissue damage, infection, or inflammation) (1). Chronic pain does not necessarily restrict to one particular location or origin. Instead, chronic pain commonly expands diffusely throughout the body (2) translating into severe levels of disability and suffering for individuals (3). In this review, we focus mainly on chronic primary pain, which includes chronic widespread pain (fibromyalgia), chronic primary headaches, or orofacial pain disorders (4). Their often bilateral manifestation may stem from central sensitization related to augmented central processing, decreased inhibition of painful stimuli or both despite no clear link to inflammation or nerve damage (5). One of the most common and impactful pain disorders is migraine, which can progress to more than 15 headache days per month referred to as chronic migraine. Although headache localized to one side is a key clinical feature of migraine, at least one-third of patients have bilateral headache (6), and it occurs more frequently in patients with near-daily attacks (25 or more days/month) compared to other chronic migraineurs (7).

When widespread pain and headaches experiences become more severe and frequent, they tend to also be associated with multiple comorbidities that are detrimental to the patients' quality of life (e.g., emotional and social dysfunction). This worsens the risk for polypharmacy and medication overuse that iatrogenically can lead to serious adverse effects like opioid addiction (8, 9). Hence, non-pharmacological pain therapies may not only be a safer option but also, most importantly, a practical pathway to scale back medication misuse in highly impacted pain patients. Neuroimaging research has elucidated particular brain regions and systems directly or indirectly associated with pain processing and analgesia. The primary motor cortex (M1) has been frequently investigated as a potential cortical target for pain relief (10). When analyzing pain neuroimaging data, researchers commonly flip the brains of the patients to match the cortical sides contralateral to the worst pain, independent of the ipsilateral response. Hence, many studies have over-emphasized the role of the most affected cortical sides. While neuromodulatory M1 protocols have been refined over decades with encouraging clinical outcomes (11), ongoing research is directed toward developing more precise technologies and enhancing consistency. We noticed two main factors limiting effectiveness and reliability in M1 pain neuromodulation when translating directly from the laboratory to the clinical setting, (1) the unilateral delivery of M1 stimulation invasively and non-invasively; and (2) for those approaches requiring hospital and laboratory-based application, the total number of sessions that can be practically delivered non-invasively.

A range of neuromodulation technologies have been established or investigated for pain management. We focus here on those that apply electrical stimulation to M1. Invasive approaches use implanted electrodes, while non-invasive approaches apply stimulation transcranially.

Non-invasive approaches include transcranial magnetic stimulation (TMS) and transcranial electrical stimulation (tES). The most common form of tES trialed for pain is transcranial Direct Current Stimulation (tDCS). tES/tDCS can be delivered using pad (sponge) electrodes (12), with the intervention typically described by the positioning of the two electrodes [e.g., M1—supra-orbital (SO)]. tES/tDCS can alternatively be provided through smaller gel-based “High-Definition” (HD) electrodes (13, 14), with the intervention typically described by the configuration of the arrays of HD electrodes [e.g., 4 × 1 (15, 16), 2 × 2 (17)]. In this review, we follow standard tES nomenclature (18), including “lateralized” for approaches where hemispheres receive symmetric but opposite polarity stimulation but adopt “diffuse asymmetric bilateral” where only one motor region is targeted. Non-invasive “unilateral focal” (sparring the opposite hemisphere) stimulation can be achieved with specific HD or TMS approaches. “Bilateral excitatory” stimulation thus refers to a particular case where motor regions in both hemispheres are excited (e.g., with same waveform/polarity) with a symmetric montage.

Current M1 neuromodulation techniques primarily target the contralateral side of dominant pain, which is rational when addressing focused neuroscientific-driven hypotheses (e.g., what waveform produces short-term changes in experimental motor excitability). Applying neuromodulation protocols developed based on experimental unilateral modulation of M1 excitability to pain management is a rational starting point, but arguably is not optimized to broad pain indications. Specifically, this raises the question of relying solely on unilateral stimulation for deriving critical changes required for satisfactory clinical outcomes and reverting or ameliorating concurrent hyper-excitability, especially in patients with having bilateral or widespread nociplastic pain as in chronic migraine or fibromyalgia (5). Moreover, it prompts us to hypothesize whether we could see differential clinical outcomes by comparing bilateral and unilateral M1 stimulation protocols by pain side/location, and global pain.

Here, we overview the most employed M1 neuromodulation methods, including TMS and tDCS. Then, we suggest strategies to improve the clinical efficacy and feasibility for pain management by introducing (1) scientific rationale/computational modeling of tDCS with enhanced focality over “bilateral” excitatory M1^2^ HD-tDCS; and (2) home-based focused M1^2^ tDCS, which enables an increasing number of session for extended treatment and replaces laboratory or clinic settings.

### Neuromodulation Approaches for Pain Modulation

We briefly summarize common neuromodulation techniques applied to acute or chronic pain by categorizing the stimulation methods into unilateral, bilateral bipolar, and bilateral excitatory stimulation (Table 1). This categorization here considers: (a) conventional tDCS approaches using diffuse asymmetric bilateral montages (e.g., M1-SO), (b) unilateral focal stimulation with invasive or non-invasive techniques (e.g., TMS); and (c) approaches producing bilateral symmetric excitation. We confine our review to studies utilizing non-invasive transcranial techniques such as tDCS and TMS, and invasive intracranial techniques such as motor cortex stimulation (MCS).

**Table 1:** Summary of brain stimulation techniques for pain improvement targeting the motor strip.

**Techniques**			**Pain type (example references)**	**Electrodes/** **modality**	**Cortical target**	**Feasibility at home**	**Non-surgical**	**Non-significant risk**	**Bilateral bipolar M1**	**Bilateral excitatory M1**
Invasive	MCS	Bipolar iMC	• Thalamic pain (19)	Permanent four array electrode	Left motor cortex; Contralateral to pain	N	N	N	N	N
			• Deafferentation pain (20)							
		Bilateral symmetric	• Dysesthetic pain (21)		Bilateral motor cortex	N	N	N	N	Y
			• Deafferentation pain (22)							
Non-Invasive	rTMS	High-frequency rTMS	• Migraine (23–26)	Figure-of-eight coil	Left motor cortex; Right motor cortex; Motor cortex contralateral to pain	N	Y	N	N	N
			• Chronic facial pain (27)							
			• Mild traumatic brain injury related headaches (28)							
			• Central Neuropathic Pain (29)							
			• Hemichorea—pain in left shoulder (30)							
			• Central pain in spinal cord injury (31)							
			• Fibromyalgia (32, 33)							
			• Central and phantom limb pain (34)							
			• Chronic neurogenic pain (35)							
		Low-frequency rTMS	• Phantom limb pain (36)	Figure-of- eight coil	Left motor cortex; Motor cortex contralateral to pain	N	Y	N	N	N
			• Chronic neuropathic pain (37)							
			• Central pain (38, 39)							
			• Deafferentation pain (40)							
		Deep rTMS	• Diabetic neuropathy (41)	Hesed (H)-coil	Lower limb region of the motor cortex	N	Y	N	Y	N
			• Neuropathic pain (42)							
	tDCS	Conventional 1 × 1 tDCS	• Central Pain in traumatic spinal cord injury (43)	Direct current electrodes with saline-soaked sponges	Left motor cortex (M1-SO montage); Left and right motor cortex for bilateral bipolar	Y	Y	Y	Y	N
			• Knee osteoarthritis (44)							
			• Fibromyalgia (45)							
			• Migraine (46, 47)							
			• Post-stroke chronic limb pain (48)							
		4 × 1 HD-tDCS	• Fibromyalgia (49, 50)	Ag/AgCl sintered ring electrodes	Left motor cortex	N	Y	Y	N	N
		2 × 2 HD-tDCS	• Chronic myofascial TMD pain (17)		Right motor cortex	N	Y	Y	N	N
		2 × 2 Bilateral M1 HD-tDCS^*^	• Migraine	4cm × 1cm strip electrodes	Left and right motor cortex	Y	Y	Y	Y	Y
			• Widespread pain such as fibromyalgia							

*HD, high-definition; iMC, ipsilateral motor cortex; MCS, motor cortex stimulation; M1-SO, primary motor cortex and contralateral supraorbital area; rTMS, repetitive transcranial magnetic stimulation; tDCS, transcranial direct current stimulation*.

* *Proposed home-based non-invasive HD-tDCS montage for bilateral M1 stimulation*.

#### Diffuse Asymmetric Bilateral

Transcranial direct current stimulation (tDCS) delivers a few milliampere (mA) of electrical stimulation across the scalp to modulate brain excitability, with the predominance of neurophysiological evidence in fact for M1 neuromodulation (51). A single session produces lasting changes that can be prolonged with repetitive (daily) tDCS applications (52). tDCS requires two electrodes (one anode and one cathode) with terminology referring only to the presumed electrode of interest (18), e.g., anodal tDCS of M1. Nonetheless, the “return” electrode remains active, and its placement (typically on the contralateral hemisphere) is presumed to create lateralized stimulation. Generally, and for our purposes, unilateral M1 tDCS refers to the placement of the “return” electrode not on the contralateral motor region, but rather typically over the contralateral supraorbital region. M1 tDCS has been reported to provide pain relief, especially to migraine [see the reviews of (53, 54)], temporomandibular disorders (17), and cancer patients (55–57), and has even shown immediate activation of the opioid system like MCS (58).

Accumulating evidence suggests the diffuse asymmetric bilateral tDCS targeting M1 is a promising tool for modulating pain, including after-surgery pain and chronic pain disorders such as fibromyalgia, neuropathic pain, phantom pain, and migraine. For instance, tDCS intervention significantly decreased patient-controlled opioid usage compared to sham after total knee arthroplasty (59) or lumber surgery (60). In fibromyalgia patients, M1 tDCS yielded more beneficial effects than dorsolateral prefrontal cortex (DLPFC) tDCS in terms of pain relief or quality of life (45) and a longer-lasting clinical efficacy of up to 1–2 months after the tDCS (61). tDCS can also potentially be used in chronic migraine showing efficacy as a preventive treatment (46). In that study, there was a significant reduction in pain intensity and length of migraine episodes after ten sessions of tDCS treatment. Moreover, pain levels continued to decrease as far as 120 days after the end of treatment.

While diffuse asymmetric bilateral M1 tDCS has a great potential in modulating pain, heterogeneity across the studies partly due to a lack of standardized protocols, and in some cases limited sample sizes, short-term treatment, and follow-up days (62), should be addressed as part of ongoing optimization and validation efforts.

#### Unilateral Focal M1 Stimulation—Invasive (MCS) and Non-invasive (TMS and HD-TDCS)

Invasive MCS has been investigated since the early 1990s as a treatment of last resort for patients with refractory pain (19). Despite some side effects (e.g., infection or hardware dysfunction) and controversies regarding efficacy (63), the surgical placement of epidural electrodes in the M1 has provided pain relief for patients with chronic neuropathic pain of various different locations and origins (64, 65), typically unilateral. For this reason and because it is highly invasive, MCS is usually restricted to the modulation of M1 contralateral to the pain side, or worse side when bilateral. In fact, scientific reports of bilateral MCS are scarce. When non-invasive technologies were developed, they tentatively mirrored MCS as a guide for their protocols, and thus adopted largely unilateral approaches. Non-invasive approaches similarly adopted unilateral stimulation for reasons of technical or protocol expediency. A further reason for non-invasive approaches employed unilateral stimulation is that they were followed by classical experimental protocols to modulate M1 with scientific rigor (66). It is thus important to note that approaches using unilateral M1 stimulation were not explicitly rationalized for the clinic under the assumption that *just* unilateral stimulation would be superior in bilateral pain disorders.

TMS in a non-invasive technology initially developed specifically to allow tolerated supra-threshold stimulation of the motor cortex (67), with repetitive protocols (rTMS) producing increased motor cortex plasticity of relevance to treatments (68–70). The ability to direct TMS for targeted cortex stimulation has been a long-standing focus, such as the development of figure-of-eight coils (71). Thus, the application of rTMS for pain treatment through M1 stimulation almost exclusively adopted focal approaches that were inherently unilateral.

Compared to conventional tDCS with the anode over M1 and the cathode over the contralateral supraorbital area, high-definition (HD)-tDCS has been more recently developed to increase focality at the target area. With HD electrodes arranged in arrays, researchers have greater flexibility to modulate excitability than in conventional tDCS (72). This unilateral focal M1 tDCS can precisely target the homuncular M1 face and head area (17, 73), hand area (74), and lower limbs (75) based on pathological brain state and study purposes. In this way, researchers can optimize the effect with flexible use of HD electrodes (smaller than conventional, <5 cm^2^), which overcomes the low spatial resolution of conventional tDCS (14, 16, 73, 76). In addition, HD-tDCS was more efficient in inducing longer-lasting neuroplasticity than conventional tDCS (16). A common HD montage is 4 × 1 HD-tDCS with one electrode surrounded by four electrodes with opposite polarity, producing focal unilateral cortical stimulation (15, 16, 77). The clinical application of 4 × 1 HD-tDCS to fibromyalgia patients has shown to reduce pain intensity in different studies (49, 50). Approaches using just 2 HD electrodes for specific unilateral M1 modulation have also been developed (78, 79) for behavior and cortical excitability examination.

HD-tDCS with a 2 × 2 montage (2 anodes and 2 cathodes) over the unilateral M1 has been tested on a selected cohort of patients with chronic TMD patients during 5-daily sessions (17, 80). The M1 chosen was contralateral to the worst TMD pain side. There were differences in sensorimotor measurements between the active and sham groups, including the pain visual analog scale (VAS). Compared with the placebo group, the active group had more responders in general pain relief (>50% in the VAS) at a 1-month follow-up. Most importantly, there was improvement of the contralateral sensory-discriminative pain measures [e.g., pain intensity, area, and their summation (PAINS)] during the treatment week, but not ipsilateral pain measures, suggesting a unilateral and focused M1 HD-tDCS stimulation selectively improved contralateral sensorimotor function.

Despite encouraging (ongoing) trails of neuromodulation targeting M1 pain, outcomes are mixed (62, 81–83). We believe optimization of dose to be key, which includes both guiding current to the brain based on rational strategies and developing protocols that allow extensive sessions—the headgear developed here supports these aims. However, the clinical effectiveness of a given dose (compared to others) can only be validated through clinical trials. They are encouraging computational modeling (76), clinical neurophysiology (78, 79), and early clinical trials (46) supporting the potency of our principled approach.

#### Bilateral M1 Excitation

In a case study of invasive bilateral stimulation over the M1 in a single patient with central dysesthetic pain and intentional tremor, most pain-evoked phenomena and improved steady burning pain and tremor were eliminated 2 month after permanent placement of MCS (21). Another single case study showed pain relief with bilateral MCS applied for deafferentation pain following spinal cord injury, suggesting that bilateral MCS could be a potentially useful treatment option for deafferentation pain (22).

There are few studies that use non-invasive stimulation over bilateral M1 for pain relief in chronic pain. Onesti et al. (41) studied the effect of 5 days of rTMS on pain relief in 23 diabetic neuropathic patients with Hesed coil (H-coil), which stimulates deeper cortical area bilaterally (41). They showed that active rTMS produced more significant pain reduction which lasted 3 weeks than sham stimulation. In a later study conducted by Shimizu et al. (42), they examined the efficacy of five daily sessions of rTMS with H-coils for lower limbc neuropathic pain, showing pain reduction 1-hour after rTMS stimulation (42). This analgesic effect did not last for more than 2 weeks, suggesting a need for revised protocols (e.g., increased number of sessions) to deliver long-term benefit to patients.

Bilateral HD-tDCS stimulation may be an effective approach for chronic pain based on recent work in healthy participants. For instance, bilateral anodal tDCS over the tongue M1 in healthy individuals induced more enhanced cortical excitability and tongue motor function than unilateral stimulation (84). Also, in a later study with healthy individuals, multifocal tDCS with bilateral M1 as anodes reverted an inhibited corticomotor excitability and impaired conditioned pain modulation that had been induced by topical experimental pain (85).

It is known that tDCS modulates resting membrane potential excitability, which outlasts a few hours or more, inducing neuroplasticity as evidenced by neurochemical or blood-oxygen-level-dependent (BOLD) signal changes in a number of cortical or subcortical regions (58, 86–91). Patients with chronic pain exhibit altered brain excitability which contributes to central sensitization features (e.g., augmented pain processing or deficient inhibitory pain modulation) (92). Neuroimaging evidence suggested that M1 tDCS with conventional montages modified μ-opioid receptor availability (58), Glx (glutamate and glutamine) level (87), or BOLD signal responses (90) of the descending pain modulatory pathway, including periaqueductal gray and anterior cingulate cortex, associated with pain sensitivity or secondary hyperalgesia. We observed that HD-M1 tDCS, in particular, reduced the contralateral side of TMD pain (17). We speculated that this analgesic effect would be delivered through the indirect/direct modulation of the ipsilateral side of the brain with highly-focused stimulation (16, 73).

Considering that precisely targeted tDCS over unilateral M1 for pain worked to partly echo how invasive MCS modulates central pain (locked into unilateral M1), it would be worth examining the effect of bilateral HD-tDCS effect on chronic pain to see if it maximizes the effect of unilateral stimulation.

## Value of Home-Based HD-TDCS

Multiple daily visits to a treatment center to participate in a study is not only burdensome for patients, but can pose a critical barrier, especially for those with disabilities or limited access to study sites. Chronic pain requires long-term management; thus, accessibility, which helps patients adhere to the treatment protocol, is critical for achieving long-term changes in neural excitability and connection. In addition, tDCS effects have been shown to be optimized with new montages, more frequent number of session or longer treatment duration, as in studies lasting over 4 weeks (17, 50, 54, 61, 93–95). If these therapeutic approaches are implemented properly at home, these efforts could significantly increase and accelerate the clinical effect reported in tDCS trials.

To date, only a few studies have shown the clinical efficacy and feasibility of home-based tDCS in certain conditions (e.g., auditory hallucination, tinnitus, and multiple sclerosis) with devices and instruction for self-administration while remotely monitoring the use of tDCS (96–98). In addition, while a few studies were conducted for pain management, recent home-based neurostimulation for the treatment of fibromyalgia has shown its feasibility and effectiveness in extending the treatment period (up to 60 sessions) and level of pain reduction (99). In another home-based study using real-time monitoring in older people with knee osteoarthritis, the patients benefited from the 10 home-based tDCS sessions through real-time monitoring in terms of reduction in pain severity and sleep disturbances (100).

There is accumulating evidence of rTMS efficacy for managing post-operative pain and chronic pain has shown promise; however, from a practical standpoint, tDCS has specific advantages over TMS, in particular the tolerance to head movement, noise, complications (e.g., risk of seizure), and price. More importantly, deployability in settings like the primary care facility, hospice (101), or home (102), especially amid a pandemic, provides a critical accessibility advantage for those already burdened by disease.

## Computational Models and Model-Driven Design of New Idea

### Computational Modeling of Bilateral M1 HD-TDCS

An individualized high-resolution finite element (FE) head model was developed from the T1-weighted MRI scan (1 mm^3^) of an adult healthy male using an automated segmentation algorithm within Statistical Parametric Mapping (SPM8, Wellcome Centre for Human Neuroimaging, London, UK). Additionally, in-house MATLAB scripts (ROAST) were used to smooth artifacts and remove discontinuities from the six different segmented image masks generated within SPM8 (103). Manual segmentation was then performed in ScanIP (Simpleware Ltd, Exeter, UK) to separate out fat from the automatically segmented image mask of skin. Stimulation electrodes, sponge pads, and gels were modeled in SolidWorks (Dassault Systèmes Corp., Waltham, MA) and imported into ScanIP for meshing. The meshes were then imported into an FE package (COMSOL Multiphysics 4.3, COMSOL, Inc., Burlington, MA, USA). Following isotropic electrical conductivities were assigned to the corresponding tissue layers and electrodes in S/m: skin: 0.465, Fat: 0.025, skull: 0.01, CSF: 0.85, gray matter: 0.276, white matter: 0.126, air: 10^−15^, electrode: 5.99 × 10^7^, and conductive gel/sponge pad: 1.4. Laplace equation (∇ · (σ∇*V*) = 0) was solved (104) and boundary conditions were used such that the current density corresponding to 2 mA of total current was applied at the anode(s) on each side (left/right M1 cortices). The ground was applied to the cathode(s). The FEM model was used to predict and compare the cortical brain current flow patterns between two different stimulation montages, 12 mm HD disk (Lab-based bilateral excitation—primary and right motor cortex) and 4 cm × 1 cm electrode strip (Home-based bilateral excitation—primary and right motor cortex). Our analyses here address current targeting, and the technology we propose can be implemented with a selection of intensity and duration, and also be adapted for transcranial alternating current stimulation (tACS) or other frequency based approaches.

#### Lab-Based Bilateral HD-TDCS 2 × 2

**Figure 1:**
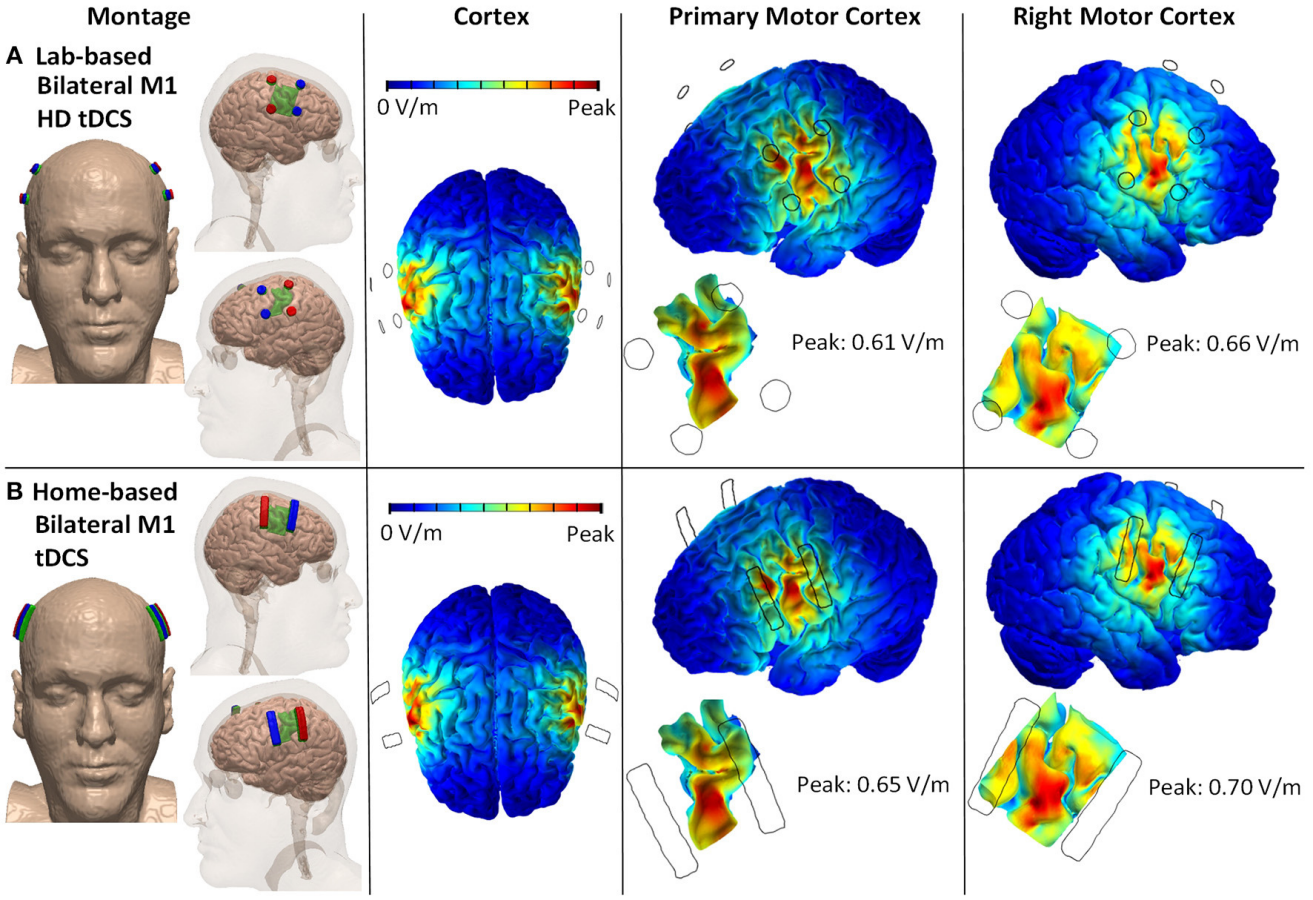
Lab- and home-based bilateral M1 HD-tDCS montages and computational modeling on 3D rendered head built from the MRI derived segmentation masks. **(A)** Lab-based with anode (red) placed over C3/C5 and cathode (blue) over FC3/FC5. **(B)** Home-based (strip) covering same regions.

Twelve millimeter diameter disk electrodes with two anodes and two cathodes positioned posterior to anterior across the face/head region of each the left and right motor cortex. Explicit 10–10 locations for the anodes are C3, C5, C4, C6, and for the cathodes are FC3, FC4, FC5, FC6 (Figure 1A).

#### Home-Based Bilateral TDCS 2 × 2

4 cm × 1 cm strip electrodes with two anodes and two cathodes positioned posterior to anterior across the face/head region of the primary and right motor cortex. Anodes are positioned horizontally across C3 and C5 and across C4 and C6. Cathodes are positioned horizontally across FC3 and FC5 and across FC4 and FC6 (Figure 1B).

The main sources for variability in the peak electric field between the two stimulation montages are differences in electrode dimensions. Home-based montage uses 4 cm × 1 cm strip electrodes, whereas the lab-based montage uses 12 mm diameter disk electrodes. The conductivity of variables (skin, fat, skull, CSF, gray matter, white matter, air, electrode, and conductive gel/sponge pad) does not vary across the two stimulation montages.

Generally, a focality of targeting or applied current intensity impacts electric field intensities. Inter-individual differences, including anatomical and functional brain state, appeared to affect the variability in the current flow in addition to the varying levels of tDCS dose (e.g., montages, current intensity) (105) thus should be understood primarily to achieve optimal outcomes.

### Development of a Home-Based HD-TDCS Headset

**Figure 2:**
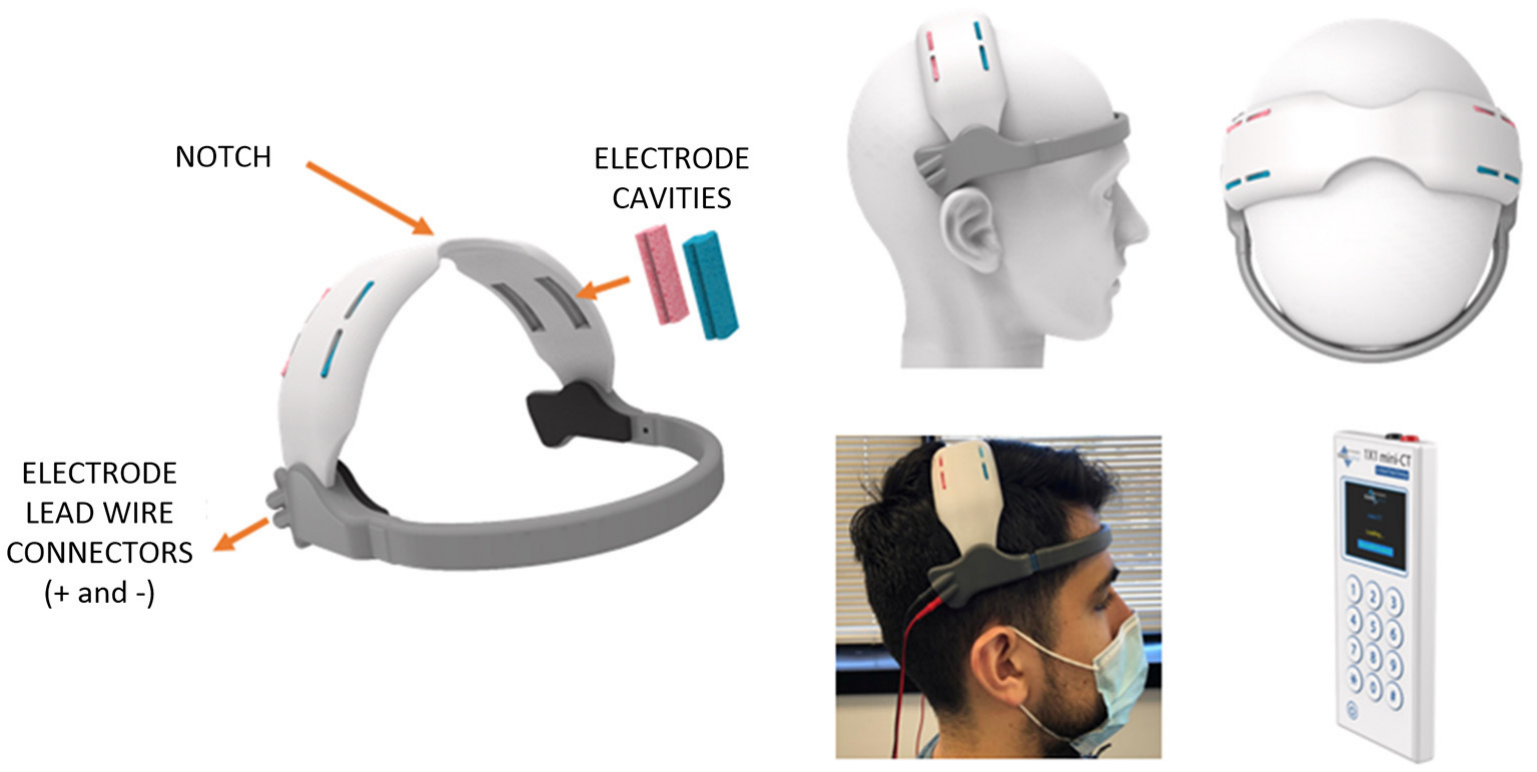
Model-driven design of home-based HD-tDCS application for bilateral M1 stimulation.

We pursued a typical iterative industrial design workflow beginning first with finalizing design constraints of the envisioned self-administered home headset. The critical functional specifications included: (1) use of a stimulation electrode solution meeting size requirement (4 cm × 1 cm), (2) need for a disposable electrode, (3) maintenance of a short anode to cathode electrode separation (replicating the distance assumed in computational model) while preventing unintended shorting, and (4) option for multiple electrode loading slots corresponding to the motor homunculus. Additionally, design requirements related to fit, usability, and aesthetics were incorporated such as secure loading, maintenance of electrode-scalp contact, subject comfort, ease of administration, and concealing of electrode leadwires. A sketch ideation phase realizing stated design constraints was pursued followed by 3D Computer Aided Design (CAD) modeling (SolidWorks, Dassault Systems SolidWorks Corp, USA). This was followed by 3D printing and associated machining to verify early concepts. These aforementioned steps were repeated until headset design was considered suitable for proceeding to the final step—i.e., suitable for loading on actual human subjects. The final design as depicted in Figure 2 consists of a forehead and a motor band. The electrode lead wires from the stimulation device are connected to the two connectors at the end of the forehead band. The electrode wires are routed within the motor band to provide the needed concealment. The forehead band includes cushioning material at different sections to help with fit and comfort. The motor band includes cavities to hold the saline-soaked single-use (disposable) sponge strips covering different levels of the homunculus. A notch is included in the center to help the user align with the nasion during the self-application process (106). A conductive carbon electrode strip is held at the base of the cavity to make the electrical connection to the sponge. Prototypes were printed using a Form 2 SLA printer (Formlabs, MA, USA) at a layer thickness of 100 microns. Though subject to feasibility clinical trials, a further principle advantage of our approach is placing electrodes across M1 will naturally drive current to (and maximize intensity at) motor cortex.

## Conclusion

Taken together, our suggested home-based HD-tDCS with enhanced focality over bilateral M1 is a highly feasible approach enabling multiple sessions at home. This claim is supported by the observation that tDCS effects are cumulative, and thus, consecutive stimulation spanning weeks to months would induce a longer-lasting impact on the neural plasticity which tDCS intends to modulate. In this manner, our new approach paves the way for novel mechanism-based treatments utilizing neuromodulation and telehealth while satisfying the compelling needs of patients and their families.

## Author Contributions

AFD, AD, and JS: design and development of home-based tDCS and analysis of data. AFD, AD, JS, DK, and MB: drafting the manuscript. All authors contributed to the article, revision for intellectual content, and approved the submitted version.

## Funding

This study was supported by grants from the National Institutes of Health — National Institute of Neurological Disorders and Stroke (NIH-NINDS R01 NS094413) and National Institute of Dental and Craniofacial Research (NIH-NIDCR U01 DE025633) United States (AD).

## Conflict of Interest

Authors AD and MB have equity in Soterix Medical Inc. The remaining authors declare that the research was conducted in the absence of any commercial or financial relationships that could be construed as a potential conflict of interest.

## Publisher's Note

All claims expressed in this article are solely those of the authors and do not necessarily represent those of their affiliated organizations, or those of the publisher, the editors and the reviewers. Any product that may be evaluated in this article, or claim that may be made by its manufacturer, is not guaranteed or endorsed by the publisher.
